# Iron and Obesity Status-Associated Insulin Resistance Influence Circulating Fibroblast-Growth Factor-23 Concentrations

**DOI:** 10.1371/journal.pone.0058961

**Published:** 2013-03-21

**Authors:** José Manuel Fernández-Real, Josep Puig, Marta Serrano, Mónica Sabater, Antoni Rubió, José María Moreno-Navarrete, Marina Fontan, Roser Casamitjana, Gemma Xifra, Francisco José Ortega, Javier Salvador, Gema Frühbeck, Wifredo Ricart

**Affiliations:** 1 Department of Diabetes, Endocrinology and Nutrition, Institut d'Investigació Biomédica de Girona, CIBERobn Fisiopatología de la Obesidad y Nutrición CB06/03/010, Girona, Spain; 2 Department of Radiology, Institut d'Investigació Biomédica de Girona, CIBERobn Fisiopatología de la Obesidad y Nutrición CB06/03/010, Girona, Spain; 3 Department of Nuclear Medicine, Institut d'Investigació Biomédica de Girona, CIBERobn Fisiopatología de la Obesidad y Nutrición CB06/03/010, Girona, Spain; 4 Department of Biochemistry, Institut d'Investigació Biomédica de Girona, CIBERobn Fisiopatología de la Obesidad y Nutrición CB06/03/010, Girona, Spain; 5 Hormonal Laboratory, CIBERdem, Barcelona, Spain; 6 Department of Endocrinology & Nutrition, Clínica Universidad de Navarra, CIBERobn Fisiopatología de la Obesidad y Nutrición, Pamplona, Spain; University of Cordoba, Spain

## Abstract

Fibroblast growth factor 23 (FGF-23) is known to be produced by the bone and linked to metabolic risk. We aimed to explore circulating FGF-23 in association with fatness and insulin sensitivity, atherosclerosis and bone mineral density (BMD). Circulating intact FGF-23 (iFGF-23) and C-terminal (CtFGF-23) concentrations (ELISA) were measured in 133 middle aged men from the general population in association with insulin sensitivity (Cohort 1); and in association with fat mass and bone mineral density (DEXA) and atherosclerosis (intima media thickness, IMT) in 78 subjects (52 women) with a wide range of adiposity (Cohort 2). Circulating iFGF-23 was also measured before and after weight loss. In all subjects as a whole, serum intact and C-terminal concentrations were linearly and positively associated with BMI. In cohort 1, both serum iFGF-23 and CtFGF-23 concentrations increased with insulin resistance. Serum creatinine contributed to iFGF-23 variance, while serum ferritin and insulin sensitivity (but not BMI, age or serum creatinine) contributed to 17% of CtFGF-23 variance. In cohort 2, CtFGF-23 levels were higher in women vs. men, and increased with BMI, fat mass, fasting and post-load serum glucose, insulin, HOMA-IR and PTH, being negatively associated with circulating vitamin D and ferritin levels. The associations of CtFGF-23 with bone density in the radius, lumbar spine and carotid IMT were no longer significant after controlling for BMI. Weight loss led to decreased iFGF-23 concentrations. In summary, the associations of circulating FGF-23 concentration with parameters of glucose metabolism, bone density and atherosclerosis are dependent on iron and obesity status-associated insulin resistance.

## Introduction

Fibroblast growth factor 23 (FGF-23) is a factor controlling inorganic phosphate metabolism and mineralization. FGF-23 is an approximately 32-kD (251 amino-acids) protein with an N-terminal region that contains the FGF homology domain and a novel 71-amino acid C-terminus. The discovery of FGF-23 revealed a tightly controlled system regulating serum phosphate. This newly discovered regulation of serum phosphate by FGF-23 is independent of PTH or the vitamin D endocrine system. Recent findings identify the skeleton as an endocrine organ and enable several abnormalities of phosphate and vitamin D metabolism to be classified as endocrine diseases [1].

Several studies have confirmed that bone is a primary site of FGF-23 production, although FGF-23 was expressed in the ventrolateral thalamic nucleus in mice [2], and weak FGF-23 expression was also observed in liver, heart, thymus and lymph nodes [3]. FGF-23 protein is detected in human bone by immunohistochemistry [4]. Recent results confirm that FGF-23 is produced by osteocytes in bone, circulates as a hormone and acts on the kidney to influence phosphate metabolism and, hence, bone mineralization [1], [5]–[8].

High-phosphate diet increases and low-phosphate diet decreases FGF-23 levels in human subjects [9]. High serum FGF-23 levels are linked to adverse outcomes such as increased mortality in patients receiving hemodialysis [10], [11] and to mortality and cardiovascular events in patients with coronary artery disease [12]. Higher FGF-23 levels, even in subjects with normal renal function, are associated with cardiovascular risk factors such as vascular dysfunction, atherosclerosis, and left ventricular hypertrophy [13]–[17]. Interestingly, circulating FGF-23 has also been recently associated with some characteristics of the metabolic syndrome in elderly individuals [18]. For this reason, we aimed to evaluate circulating intact FGF-23 (iFGF-23) and C-terminal (CtFGF-23) concentrations (ELISA) in association with metabolic parameters such as fat mass, insulin sensitivity, bone mineral density and intima media thickness. Circulating iFGF-23 was also measured before and after weight loss.

## Materials and Methods

### Cohort 1

One hundred and thirty-three subjects (all men) were randomly localized from a census and they were invited to participate. The participation rate was 71%. A 75-g oral glucose tolerance test (OGTT) according to the American Diabetes Association Criteria was performed in all subjects.

Inclusion criteria were 1) BMI<40 kg/m^2^, 2) absence of systemic disease, and 3) absence of infection within the previous month. None of the control subjects were under medication or had evidence of metabolic disease other than obesity. Liver disease and thyroid dysfunction were specifically excluded by biochemical work-up. All subjects had normal serum creatinine levels.

#### Measurements

Subjects were studied in the post-absorptive state. Body weight was measured with a digital scale to the nearest 0.1 kg, and height was measured to the nearest 0.1 cm with a Holtain stadiometer (Holtain Ltd., Crymych, UK).

Blood pressure was measured in the supine position on the right arm after a 10-min rest; a standard sphygmomanometer of appropriate cuff size was used and the first and fifth phases were recorded. Values used in the analysis are the average of three readings taken at 5-min intervals.

#### Insulin sensitivity

Insulin sensitivity was measured using the frequently sampled intravenous glucose tolerance test (FSIVGTT) on a different day. In brief, basal blood samples were drawn at –15 and –5 min, after which glucose (300 mg/kg body wt) was injected over 1 min starting at time 0. At 20 min, regular insulin (Actrapid, Novo, Denmark; 0.03 U/kg) was injected as a bolus. Additional samples were obtained from a contralateral antecubital vein at times 1, 2, 3, 4, 5, 6, 7, 8, 10, 12, 14, 16, 19, 20, 22, 23, 24, 25, 27, 30, 40, 50, 60,70, 80, 90, 100, 120, 140, 160, and 180 min. Samples were rapidly collected via a three-way stopcock connected to a butterfly needle. Data from the FSIVGTT were submitted to computer programs that calculate the characteristic metabolic parameters by fitting glucose and insulin to the minimal model that describes the times course of glucose and insulin concentrations. The glucose disappearance model, by accounting for the effect of insulin and glucose on glucose disappearance, provides the parameters SI (10–4) per minute per microunit per milliliter) or the insulin sensitivity index, a measure of the effect of insulin concentrations above the basal level to enhance glucose disappearance. The estimation of model parameters was performed according to the MINMOD computer program [19], as previously described [20].

### Cohort 2

From January 2010 to February 2012, we consecutively recruited subjects from the ongoing multicenter FLORINASH Project. Inclusion criteria were age 30 to 65 years, body mass index (BMI)>30 kg/m^2^, and ability to understand study procedures. Exclusion criteria were systemic disease, infection in the previous month, serious chronic illness, >20 g ethanol intake per day, or use of medications that might interfere with insulin action. Control non-obese men and women were also included. The institutional review board approved the study protocol, and all subjects provided informed written consent.

#### Study Protocol

Each patient underwent anthropometric measurements, vascular and abdominal ultrasound, and laboratory parameters on the same day. After 8 h fasting, blood was obtained for measurement of plasma lipids, glucose, and insulin. Glucose and lipid levels were determined by standard laboratory methods. Serum insulin was measured by radioimmunoassay, as previously described [20]. Insulin resistance was determined by the homeostasis model assessment of insulin resistance (HOMA-IR).

#### Body Fat and bone mineral density

Fat mass and bone mineral density were determinated by dual energy x-ray absorptiometry (DEXA), using a Lunar Prodigy Full Oracle (GE Healthcare, enCore software version 13,2). Whole body composition (fat mass, fat-free soft tissue mass and bone mineral content) was obtanined according to standard procedures, by trained personnel. Bone mineral density was measured in lumbar spine (L1–L4) and in distal radius (radius UD), using Word Health Organization T-score criteria for Caucasian population [21].

#### Ultrasound Evaluation

We used a Siemens Acuson S2000 (Mochida Siemens Medical System, Tokyo, Japan) ultrasound system with a 3.5 MHz convex transducer to scan the liver and a 7.5 mHz linear array transducer to scan carotid arteries. Images were transferred to Starviewer software, developed in our laboratory (http://gilab.udg.edu), and independently evaluated by two radiologists blinded to clinical and laboratory data.

Carotid arteries were evaluated according to the Mannheim Consensus [22]. c-IMT values were manually measured in the far wall of each common carotid artery in two locations a) in a proximal segment and b) in a plaque-free segment 10 mm from the bifurcation. The mean c-IMT value for each subject was calculated from these four measurements.

### Study of the effects of weight loss

An independent cohort composed of 10 Caucasian obese men attending the *Endocrinology Department at the University Clinic of Navarra* was recruited. Weight loss was achieved by prescription of a diet providing a daily energy deficit of 500–1000 kcal/d as calculated from the determination of the resting energy expenditure through indirect calorimetry (Vmax29, SensorMedics Corporation, Yorba Linda, California) and multiplication by 1.4 as indicated for sedentary individual's to obtain the patient's total energy expenditure. This hypocaloric regime allows a safe and steady weight loss of 0.5–1.0 kg/wk when followed and supplied 30, 54 and 16% of energy requirements in the form of fat, carbohydrates and protein, respectively.

#### Ethics statement

The institutional review board, Comité d'Ètica d'Investigació Clínica (CEIC) from the Hospital de Girona "Dr Josep Trueta" approved the study protocol, and all subjects provided informed written consent.

#### Analytical methods

Serum glucose concentrations were measured in duplicate by the glucose oxidase method using a Beckman glucose analyser II (Beckman Instruments, Brea, California). Intraassay and interassay coefficients of variation were less than 4% for all these tests. HDL cholesterol was quantified after precipitation with polyethylene glycol at room temperature. Total serum triglycerides were measured through the reaction of glycerol/phosphate/oxidase and peroxidase. Serum insulin was measured in duplicate in the same centralized laboratory by a monoclonal immunoradiometric assay (Medgenix Diagnostics, Fleunes, Belgium). The intra-assay coefficient of variation (CV) was 5.2% at a concentration of 10 mU/l and 3.4% at 130 mU/l. The inter-assay CVs were 6.9 and 4.5% at 14 and 89 mU/l, respectively. Osteocalcin was measured by an Enzyme Amplified Sensitivity Immunoassay (EASIA) kit (DRG Instruments GmbH, Marburg, Germany). Sensitivity of the method, the detection limit, defined as the apparent concentration two standard deviations above the average OD at zero binding, was 0.4 ng/ml and the intra- and inter-assay CV were less than 10%.

FGF23 level in serum was measured by a commercial ELISA Kit (FGF-23 Elisa Kit; Kainos Laboratories, Inc, Japan) in samples that were conserved using a protease inhibitor, according to the manufacturer's protocol. The intra-assay coefficient of variation was between 2.0 and 3.0% and the inter-assay coefficient of variation was between 2.0 and 3.8%. CtFGF23 levels were measured using a commercial ELISA kit (Human FGF-23 (C-term) ELISA Kit; Immutopics, Inc; San Clemente, CA) in EDTA plasma. The intra-assay CV was between 1.4% and 2.4% and the inter-assay CV was between 2.4% and 4.7%. All FGF23 measurements were centralized in a single laboratory.

#### Statistical methods

Statistical analyses were performed using SPSS 12.0 software. Unless otherwise stated, descriptive results of continuous variables are expressed as mean and SD for Gaussian variables. Parameters that did not fulfill normal distribution were logarithmically transformed to improve symmetry for subsequent analyses. The relation between variables was analyzed by simple correlation (Pearson's test). Multiple linear regression analyses were performed in a stepwise manner to predict circulating FGF-23 concentration. Levels of statistical significance were set at P<0.05.

## Results

**Figure 1:**
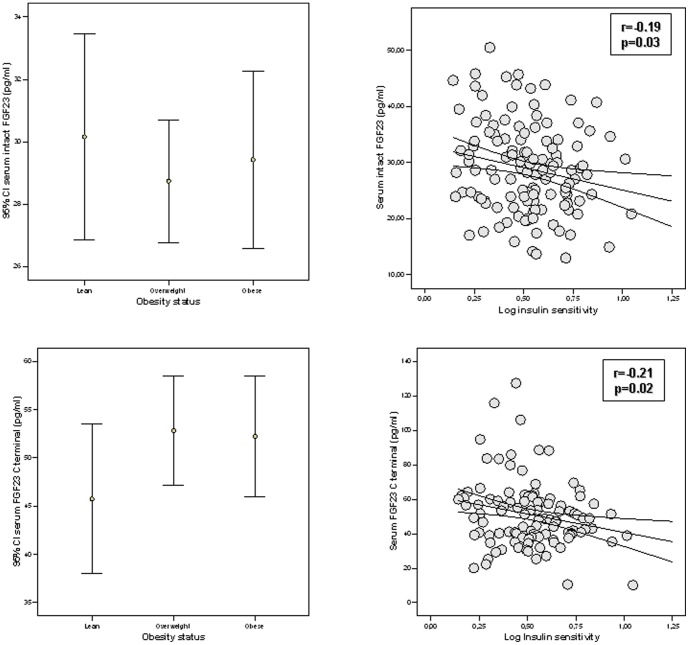
FGF-23 associations with obesity. **a)** Linear association analysis between serum intact FGF-23 concentrations and body mass index; **b)** serum intact FGF-23 concentration according to obesity status; **c)** Linear association analysis between serum C-terminal FGF-23 concentrations and body mass index; **d)** serum C-terminal FGF-23 concentration according to obesity status. All subjects from both cohort 1 and 2 are included.

Anthropometrical and biochemical characteristics of subjects included in cohort 1 and 2 are shown in Table 1 and 2, respectively. We first explored the possible association of serum FGF-23 concentrations with obesity. In all subjects as a whole, serum intact and C-terminal concentrations were linearly and positively associated with BMI and increased with obesity status (Figure 1).

**Table 1:** Anthropometrical and biochemical characteristics of the study subjects in Cohort 1.

n	133 men
Age (years)	51.8±11.6
BMI (kg/m^2^)	27.6±3.5
Waist perimeter (cm)	92.6±9.8
Systolic blood pressure(mmHg)	127.7±14.8
Diastolic blood pressure (mmHg)	80.5±9.6
Cholesterol (mg/dl)	213.9±38.01
LDL-cholesterol (mg/dl)	138.08±35.2
HDL-cholesterol (mg/dl)	53.1±12.2
Log fasting triglycerides (mg/dl)	1.97±0.24
Fasting glucose (mg/dl)	97.5±11.2
Post-load glucose OGTT (mg/dl)	135.1±45.7
Post-load insulin OGTT (mU/L)	70.4±63.7
Log Insulin sensitivity (10^−4*^min^−1*^mU/L)	0.4±0.2
Log serum ferritin (ng/ml)	2.09±0.35
Creatinine (mg/dl)	1.01±0.1
PTH (pg/ml)	44±14.4
Vitamin D (ng/ml)	21.8±6.5
Serum phosphate (mg/dl)	3.93±0.69
iFGF-23 (pg/ml)	28.8±7.8
Ct FGF23 (pg/ml)	51±20.7

**BMI**, Body mass index; **CRP**, C reactive protein, **iFGF-23**, fibroblast growthfactor-23, **CtFGF-23**, C-terminal FGF-23 **OGTT**, oral glucose tolerance test; **PTH**, parathyroid hormone.

**Table 2:** Anthropometrical, clinical and biochemical characteristics of the study subjects in Cohort 2.

	Men	Women	`p
n	262	52	-
Age (years)	42.2±7.4	40.8±8.6	0.4
BMI (kg/m^2^)	34.1±9.5	33.3±10.6	0.7
Waist perimeter (cm)	107.8±21.8	96.1±21.2	0.02
Fat mass (kg)	38.9±20.5	40.3±18.7	0.7
Systolic blood pressure(mmHg)	136±18.5	127.8±17	0.05
Diastolic blood pressure (mmHg)	78.1±11.1	71±10.1	0.006
Right ICAIMT (mm)	0.58±0.12	0.53±0.15	0.2
Left ICAIMT (mm)	0.62±0.12	0.54±0.14	0.02
Radium T-score	0.7 (−1–1.7)	0.35 (−0.3–1.5)	0.3
Lumbar T-score	−0.8 (−1.2–0.42)	0.35 (−1–1.7)	0.02
Cholesterol (mg/dl)	192.4±30.6	197±38.7	0.6
LDL-cholesterol (mg/dl)	121.4±26.6	116.3±33.9	0.5
HDL-cholesterol (mg/dl)	45.8±13.2	61.6±19.3	<0.0001
Log fasting triglycerides (mg/dl)	2.02±0.2	1.93±0.19	0.07
Fasting glucose (mg/dl)	92.1±11.2	89.8±16.6	0.5
Fasting insulin (mU/L)	7.6 (1.8–12.2)	4.9 (2–9.9)	0.3
Post-load glucose OGTT (mg/dl)	120.4±34.9	116.1±42.9	0.6
Post-load insulin OGTT (mU/L)	52.5±43.9	44.7±32	0.4
HOMA	1.91 (0.14–4.76)	1.07 (0.41–2.08)	0.2
Log serum ferritin (ng/ml)	2.3±0.3	1.57±0.4	<0.0001
Creatinine (mg/dl)	0.82±0.14	0.66±0.11	<0.0001
PTH (pg/ml)	48.3±14.8	47.9±20	0.9
Serum phosphate (mg/dl)	3.2±0.3	3.36±0.4	0.16
Vitamin D (ng/ml)	18.3±8.39	20.3±11.7	0.4
iFGF-23 (pg/ml)	32.7±9.3	35.1±16.2	0.4
CtFGF-23 (pg/ml)	54.4±21.8	72.04±34.1	0.007

**BMI**, Body mass index; **CRP**, C reactive protein, **iFGF-23**, fibroblast growth factor-23, **CtFGF-23**, C-terminal FGF-23, **ICAIMT**, internal carotid artery intima media thickness, **OGTT**, oral glucose tolerance test; **PTH**, parathyroid hormone. Values are given as mean ± SD or as median (interquartile range).

**Figure 2:**
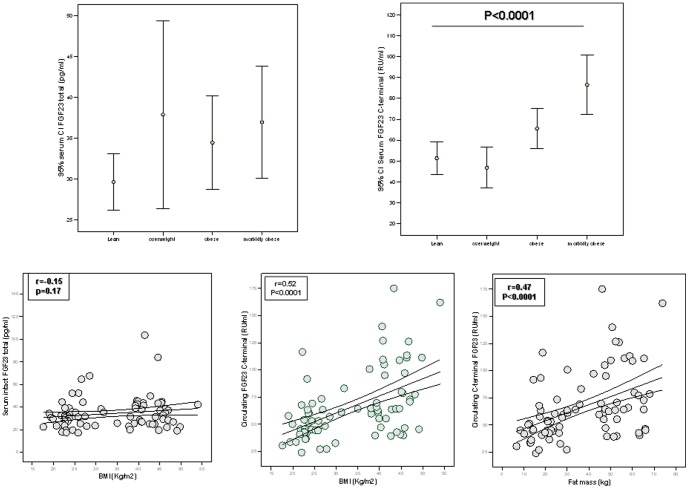
FGF-23 associations with insulin sensitivity. **a)** Linear association analysis between serum intact FGF-23 concentration and insulin sensitivity in subjects from cohort 1; **b)** Linear association analysis between serum C-terminal FGF-23 concentration and insulin sensitivity in subjects from cohort 1.

In cohort 1, both serum iFGF-23 and CtFGF-23 concentrations decreased linearly with insulin sensitivity (Figure 2 and Table 3). While iFGF-23 was associated with serum creatinine, CtFGF-23 was linked to serum ferritin concentration (r = −0.20, p = 0.02, Table 3). Multiple linear regression models were constructed to predict circulating intact or C-terminal FGF-23 levels, with BMI, age, serum creatinine, ferritin and insulin sensitivity as dependent variables. Serum creatinine emerged as the only variable that contributed to iFGF-23 variance (6%, p = 0.005) while serum ferritin (p<0.0001) and insulin sensitivity (p = 0.002), but not BMI, age or serum creatinine contributed to 17% of CtFGF-23 variance.

**Table 3:** Linear correlation analysis of the association between circulating intact and CtFGF-23 levels and selected variables in Cohort 1.

	CtFGF-23	p	Intact FGF-23	P
Age	0.06	0.4	0.13	0.12
BMI	0.17	0.06	−0.002	0.98
Creatinine	0.09	0.29	0.29	0.001
Ferritin	−0.20	0.02	0.009	0.9
Log Insulin sensitivity	−0.21	0.02	−0.19	0.03
Post-load insulin OGTT	0.23	0.01	0.19	0.03
Serum phosphate	0.17	0.06	0.19	0.03

**Figure 3:**
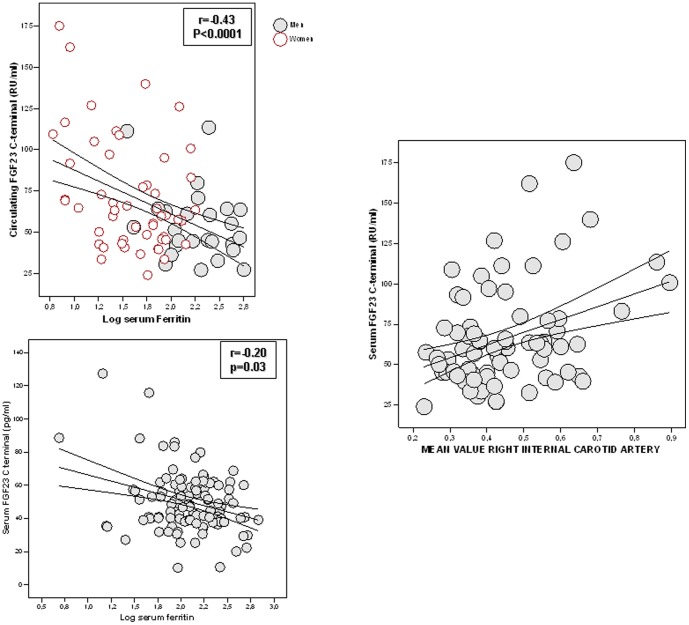
FGF-23 associations with ferritin and intima media thickness. Linear association analysis between serum C-terminal FGF-23 concentration and serum ferritin levels in Cohort 1 (**a**) and 2 (**b**); **c)**. Linear association analysis between serum C-terminal FGF-23 concentration and intima media thickness in subjects from cohort 2.

Cohort 2 included both men and women (Table 2). Serum CtFGF-23 levels were significantly increased in women vs. men (71.8±33.8 vs. 54.3±21.8, p = 0.008) despite men and women being of similar age (Table 2). In this cohort, circulating CtFGF-23 levels increased with BMI (r = 0.51, p<0.0001), DEXA-fat mass (r = 0.47, p<0.0001), fasting and post-load serum glucose (r = 0.26, p = 0.02, and r = 0.29, p = 0.01, respectively), fasting and post-load serum insulin (r = 0.38, p = 0.003, and r = 0.28, p = 0.03, respectively, Table 4) and insulin resistance (HOMA-IR, r = 0.35, p = 0.006). Circulating CtFGF-23 levels correlated positively with serum PTH (r = 0.28, p = 0.01) and negatively with serum vitamin D levels (r = −0.27, p = 0.01) and serum ferritin concentrations (Figure 3, r = −0.43, p<0.0001).

**Table 4:** Linear correlation analysis of the association between serum intact and CtFGF-23 levels and selected variables in Cohort 2.

	CtFGF-23	p	Intact FGF-23	P
Age	0.10	0.37	0.23	0.04
BMI	0.52	<0.0001	0.15	0.16
Creatinine	0.003	0.9	0.34	0.003
Ferritin	−0.43	<0.0001	0.07	0.5
HOMA	0.35	0.006	0.17	0.1
Post-load insulin OGTT	0.28	0.03	0.18	0.1
Serum phosphate	0.16	0.2	0.37	0.003

Circulating CtFGF-23 concentration was also positively associated with bone density in the radius (T-score, r = 0.23, p = 0.04) and tended to be associated with bone density at the lumbar spine (T-score, r = 0.21, p = 0.06) but these associations were no longer significant after controlling for BMI. Interestingly, CtFGF-23 concentration was also positvely associated with the mean value of the right internal carotid artery intima media thickness (ICAIMT, r = 0.36, p = 0.002, Figure 3) and of the left ICAIMT (r = 0.25, p = 0.03). Again, these associations were no longer significant after controlling for BMI.

Similarly to Cohort 1, serum iFGF-23 levels correlated significantly with serum creatinine (r = 0.34, p = 0.003) and with age (r = 0.23, p = 0.04). In addition, serum iFGF-23 levels were positively associated with serum PTH (r = 0.30, p = 0.007) and serum phosphate concentrations (r = 0.37, p = 0.003) but not with the remaining parameters depicted above (r<0.20, p>0.15).

In a multiple linear regressiona analysis, BMI (P = 0.001) and serum ferritin (p<0.0001), but not age (p = 0.26), HOMA value (0.36), creatinine (0.23) or sex (p = 0.98) contributed independently to 45% of the variance in circulating CtFGF-23 levels. On the contrary, serum creatinine (p = 0.006), age (p = 0.04), and sex (p = 0.003), but not BMI (P = 0.11), HOMA (p = 0.65), or serum ferritin (p = 0.21), contributed independently to 20% of the variance in circulating iFGF-23 levels.

### Weight loss study

**Figure 4:**
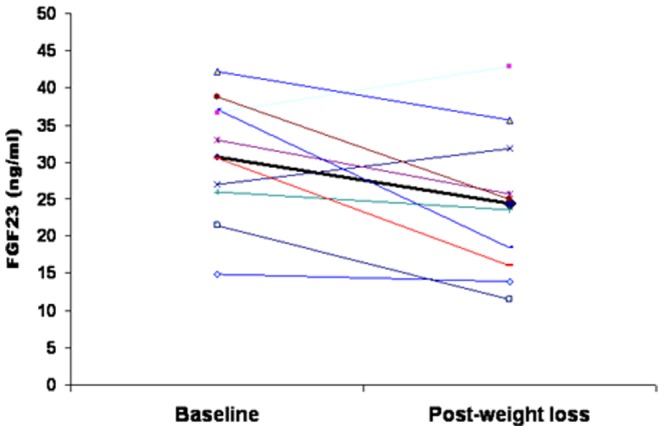
Individual changes in serum intact FGF-23 concentrations in obese subjects after weight loss.

Characteristics of the subjects are shown in Online table. Weight loss led to decreased circulating FGF-23 concentrations (Figure 4) in parallel to decreased HOMA-IR (Table 5). Post-weight loss (but not pre-weight loss) circulating FGF-23 concentrations were significantly associated with HOMA value.

**Table 5:** Subjects' characteristics in the diet-induced weight loss study (10 men, aged 43±15 years).

	Baseline	Post-weight loss	*Paired t*-test
**Weight (kg)**	**105.5±32.4**	**85.2±9.8**	**0.02**
**BMI (kg/m^2^)**	**33.8±8.4**	**27.4±1.8**	**0.01**
**Fat mass (kg)**	**37.05±6.6**	**25.8±3.3**	**0.0001**
**Waist (cm)**	**115.3±17.8**	**98.2±5.02**	**0.004**
**WHR**	**1.04±0.03**	**0.9±0.03**	**0.002**
**SBP (mmHg)**	**125.6±14.8**	**116.6±9.6**	**0.08**
**DBP (mmHg)**	**81.7±6.7**	**75±6.6**	**0.01**
**Fasting Glucose (mg/dL)**	**95.2±5.5**	**91.75±6.6**	**0.2**
**HOMA-IR**	**4.2±3.4**	**2.5±1.3**	**0.04**
**Total cholesterol (mg/dL)**	**211.1±39.4**	**178.2±21.9**	**0.04**
**HDL-cholesterol (mg/dL)**	**50.6±11.6**	**49.5±12.9**	**0.4**
**LDL-cholesterol (mg/dL)**	**134.6±34.3**	**112.7±18.05**	**0.08**
**Fasting triglycerides (mg/dL)**	**129±71.1**	**79.4±39.3**	**0.01**
**creatinine (mg/dl)**	**0.95±0.19**	**0.92±0.16**	**0.4**
**iFGF-23 (ng/mL)**	**30.7±8.4**	**24.4±10.05**	**0.03**

**BMI**, Body mass index; DBP, diastolic blood pressure, i**FGF-23**, intact fibroblast growth factor-23, **HOMA**, Homeostatic model of assessment, **SBP**, systolic blood pressure, **WHR**, waist-to-hip ratio.

## Conclusions

FGF23 principally functions as a hormone regulating phosphaturia, suggesting that this circulating factor also mediates secretion of PTH at least indirectly by regulating serum phosphate. FGF23 induces urinary phosphate excretion by suppressing the expression of sodium/phosphate cotransporter [1]. In agreement with these functions, we found that circulating iFGF-23 was positively associated with serum phosphate and PTH concentrations.

The type of information captured by the intact vs. C-terminal assay of FGF-23 seems different. The C-terminal ELISA assay can detect both the intact FGF23 hormone and the C-terminal FGF23 fragment. Some authors [23] have proposed that the C-terminal FGF23 ELISA captures better the biologically functional FGF23 molecule which is decreasing phosphate reabsorption in the renal tubules. However, it should be taken into account that C-terminal FGF23 peptides might display opposite effects. Goetz et al have described that injection of the FGF23 C-terminal tail peptide into healthy rats inhibited renal phosphate excretion and induced hyperphosphatemia [24].

According to our findings, renal function within the normal range significantly impacts on iFGF-23 concentrations, given the positive associations with serum creatinine in both cohorts, and the association with parameters that influence serum creatinine concentrations, such as age and sex (in multiple linear regression analysis of Cohort 2).

One of the most consistent findings regarding CtFGF-23 was its association with serum ferritin levels. Iron has been described to act on FGF23 pathways by inhibiting the cleavage of the intact FGF23 molecule and in assisting the clearance of FGF23 fragments by the kidney [23], [25]. Thus, the higher the iron stores, the lower the CtFGF-23 concentration. And *vice versa*, the lower the ferritin levels, the higher the CtFGF-23 levels. Iron deficiency has been described to stimulate HIF1α leading to increased FGF-23 transcription [25], [26].

We here show tha serum intact and C-terminal increase linearly with BMI and obesity status. This finding suggest that increased bone strain with increased adiposity might lead to increased secretion of FGF-23. Subjects with morbid obesity showed the highest (almost 2-fold) CtFGF-23 levels, being BMI the factor that most contributed to its variance. In agreement with this finding, some studies have shown show a positive correlation of FGF23 and body weight and fat tissue content in elderly men. For instance, a significant correlation between intact FGF23 and body weight (r = 0.13, p<0.0001) was found in 3014 Swedish men aged 69–80 years [18], [27]. In another study, participants in the highest category of BMI had 9.5 RU/ml higher C-terminal FGF23 than those in the lowest [28]. In 48 perimenopausal obese women and in 29 nonobese controls, iFGF-23 concentrations correlated significantly with BMI (r = 0.292) and fat content (r = 0.259) in all study subjects [29]. A recent case-control study was performed in 20 women having bariatric surgery and 20 control women matched for race and age. FGF23 was higher in the bariatric patients than the controls [30]. There is no mention of the assay employed. Interestingly, in experimental studies, leptin stimulated FGF23 expression in bone and suppressed renal 1alpha,25-dihydroxyvitamin D3 synthesis in leptin-deficient mice [31].

Insulin sensitivity, using a robust measurement as the minimal model method, also contributed independently to the variance of serum CtFGF-23 levels in Cohort 1 in which only men with BMI<40 kg/m^2^ and a wide range of insulin action were included. There is only one study, to our knowlege, investigating the association with insulin sensitivity. Using HOMA value, Wojcik M et al. found a preliminar association of iFGF-23 with insulin resistance in obese adolescents [32]. In line with these results, acute hyperinsulinemia has beeen described to increase serum FGF-23 concentrations in type 2 diabetes patients [33].

Thus it can be concluded that the most obese, insulin resistant subjects with low iron stores exhibit the highest FGF-23 concentrations, as measured by the C-terminal assay. It is tempting to speculate that the mechanical shear stress and loading of obesity interact with the relative hypoxia of low iron stores on bone to secrete FGF-23.

That extreme obesity was important in determining CtFGF-23 concentrations was also confirmed by the findings regarding bone density and carotid intima media thickness. Although CtFGF-23 correlated positively with these parameters, these associations were no longer significant when BMI was taken into account. In addition, weight loss led to decreased FGF-23 concentrations.

In the aforementioned study [27], there was a weak but significant correlation between intact FGF23 and bone mineral density in femoral neck (r = 0.04, p<0.05), femoral trochanter (r = 0.05, p = 0.004), total hip (r = 0.06, p = 0.0015) and lumbar spine (r = 0.07, p = 0.0004). As in the current study, the associations became insignificant in all regions when adjusting for established confounding variables including age, height and weight [27].

Circulating FGF-23 concentration has also been described to be associated with several cardiovascular risk factors and atherosclerosis [13]–[18]. We here characterize that this association depends on the FGF-23 assay and on the presence of morbid obesity.

In summary, there is a differential association of circulating FGF-23 concentration with parameters of glucose metabolism, bone density and atherosclerosis that is dependent on iron and obesity status. It remains to be determined, which signals, other than phosphate, regulate FGF23 production and which molecules mediate its regulation.
